# Measurement of the Immunosuppressant Possession Ratio by Transplant Clinical Pharmacists Captures a Non-Adherence Associated With Antibody-Mediated Rejection

**DOI:** 10.3389/ti.2023.11962

**Published:** 2023-11-28

**Authors:** Jérémy Chambord, Bertrand Chauveau, Sarah Djabarouti, Jean Vignaud, Benjamin Taton, Karine Moreau, Jonathan Visentin, Pierre Merville, Fabien Xuereb, Lionel Couzi

**Affiliations:** ^1^ Service de Pharmacie, Centre Hospitalier Universitaire de Bordeaux, Bordeaux, France; ^2^ Service d’Anatomopathologie, Centre Hospitalier Universitaire de Bordeaux, Bordeaux, France; ^3^ CNRS-UMR 5164 ImmunoConcEpT, Université de Bordeaux, Bordeaux, France; ^4^ INSERM U1312 BRIEC, Université de Bordeaux, Bordeaux, France; ^5^ Service de Néphrologie, Transplantation, Dialyse, Aphérèse, Centre Hospitalier Universitaire de Bordeaux, Bordeaux, France; ^6^ Service d’Immunologie et Immunogénétique, Centre Hospitalier Universitaire de Bordeaux, Bordeaux, France; ^7^ INSERM U1034, Université de Bordeaux, Bordeaux, France

**Keywords:** kidney transplantation, clinical pharmacist, adherence, immunosuppresant possession ratio, antibody-mediated rejection

## Abstract

Our objective was to calculate an immunosuppressant possession ratio (IPR) to diagnose non-adherence at the time of antibody-mediated rejection (ABMR). IPR was defined as the ratio of number of pills collected at the pharmacy to the number of pills prescribed over a defined period. In a first cohort of 91 kidney transplant recipients (KTRs), those with an IPR < 90% had more frequently a tacrolimus through level coefficient of variation >30% than patients with an IPR = 100% (66.7% vs. 29.4%, *p* = 0.05). In a case-control study, 26 KTRs with ABMR had lower 6 months IPRs than 26 controls (76% vs. 99%, *p* < 0.001). In KTRs with ABMR, non-adherence was more often diagnosed by a 6 months IPR < 90% than by clinical suspicion (73.1% vs 30.8%, *p* = 0.02). In the multivariable analysis, only *de novo* DSA and 6 months IPR < 90% were independently associated with ABMR, whereas clinical suspicion was not (odds ratio, 4.73; 95% CI, 1.17–21.88; *p* = 0.03; and odds ratio, 6.34; 95% CI, 1.73–25.59; *p* = 0.007, respectively). In summary, IPR < 90% is a quantifiable tool to measure immunosuppressant non-adherence. It is better associated with ABMR than clinical suspicion of non-adherence.

## Graphical Abstract

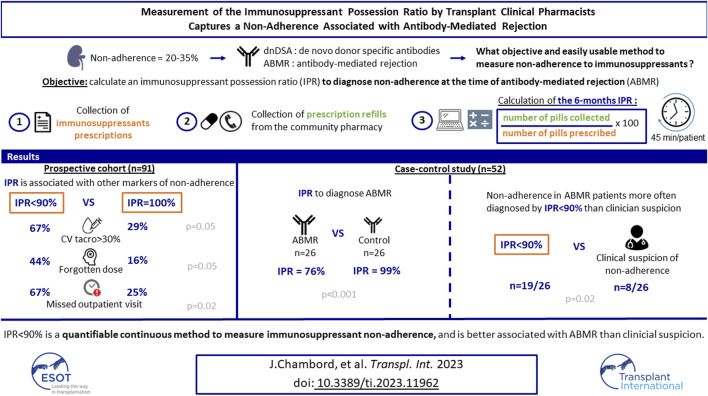

## Introduction

The prevalence of non-adherence to immunosuppressants in renal transplant recipients is between 20%–35% in adults [1–3]. This is a continuous process which increases during the first 2 years post-transplantation [1, 4, 5], and is associated with *de novo* donor-specific antibodies occurrence (DSA) [6–8], antibody-mediated rejection (ABMR) [7, 9], T-cell mediated rejection [6, 10], and graft loss [3, 11–16]. Proactive interventions to improve adherence are essential for the prevention of allograft loss. However, before designing a suitable multi-dimensional intervention, the key question is how immunosuppressant non-adherence can be diagnosed prospectively [17].

Subjective methods to assess non-adherence include clinical suspicion and self-administered questionnaires. Suspicion by the clinician of medication non-adherence underestimates this phenomenon and is frequently influenced by a poor outcome or non-adherence to follow-up [18]. Self-reported measurement of medication non-adherence is easily distorted by patients, explaining why its ability to predict rejection and graft loss is equivocal [19–23]. Objective methods for the measurement of non-adherence include calcineurin inhibitor trough levels and electronic monitoring. Both low calcineurin inhibitor trough levels [24–27] and intra-patient variability of tacrolimus are associated with *de novo* DSA, rejection, and graft loss [28–31]. However, interactions with a drug or food can give a false impression of non-adherence. The ability of electronic monitoring to measure non-adherence to immunosuppressants is also debated [17, 21, 22, 32–35]. In addition, this tool is very costly and restrictive, which could prevent its implementation in a clinical setting.

Therefore, transplant physicians do not yet have an objective and easily usable method for measuring non-adherence to immunosuppressants [1]. The Immunosuppressant Possession Ratio (IPR) is the number of therapeutic units collected at the pharmacy divided by the number of therapeutic units prescribed over the same period of time [12]. Retrospective studies using Medicare data in the United States reported that low IPRs were associated with graft failure [11, 12, 15, 36]. In these studies, IPR thresholds used to determine non-adherence varied between 80% and 99%. In France, the rate of prescription refill can be easily retrieved through pharmacy management software. No special authorization is required.

The objectives of this study were: 1) to test the feasibility of prospectively calculating the IPR in a first cohort of kidney transplant recipients (KTR), 2) to determine its association with other markers of non-adherence, 3) to determine a standardized period for its calculation, 4) to analyze whether the IPR-based non-adherence diagnosis was associated with ABMR in a second cohort of KTR, and 5) to compare the IPR-based non-adherence diagnosis with our standard method based on clinical suspicion.

## Patients and Methods

### Study Design and Patients

#### First Prospective Cohort

**FIGURE 1:**
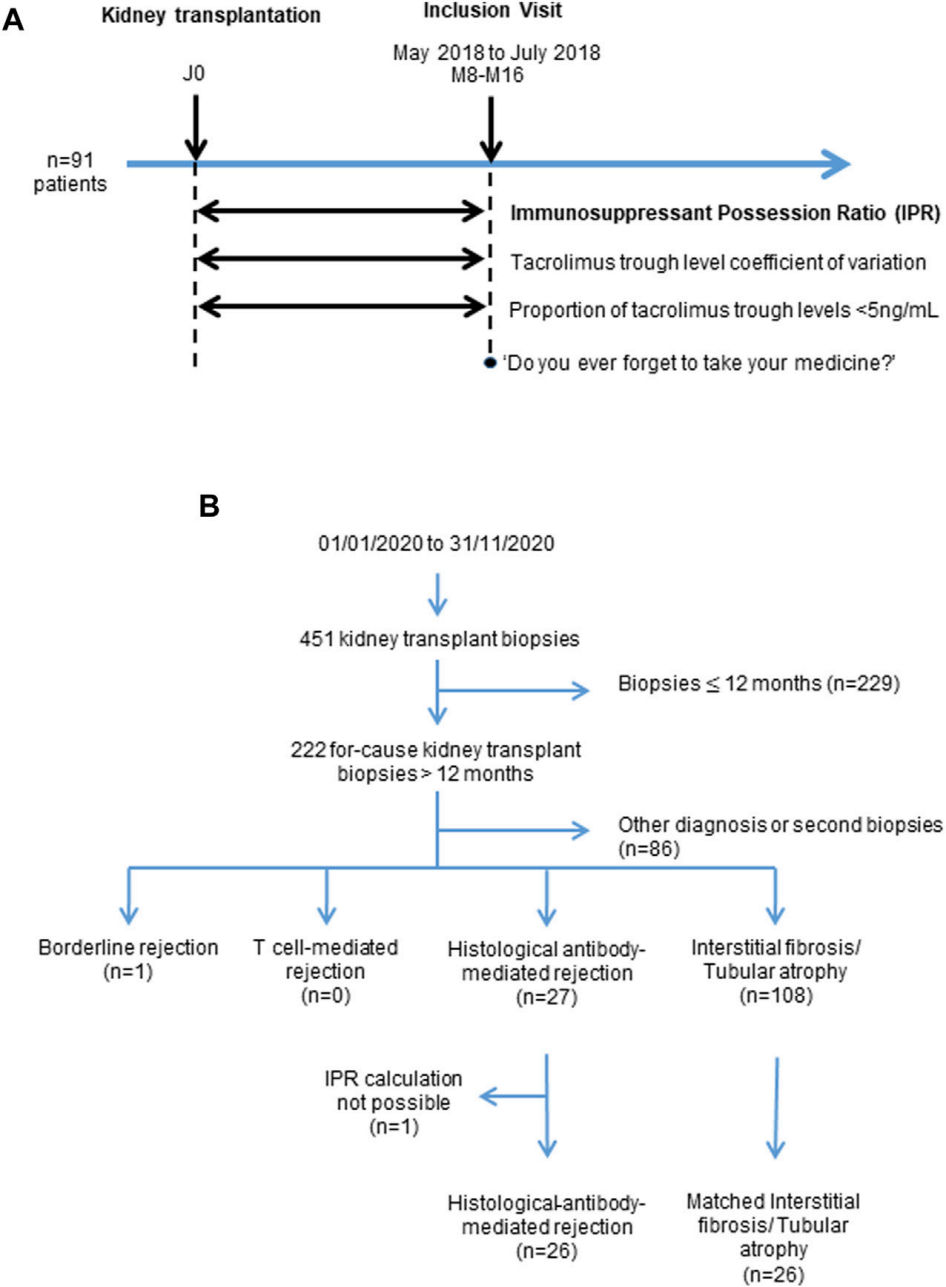
Description of the two Cohorts. Study design of the first prospective Cohort 1 **(A)**. Flow chart of the retrospective Cohort 2 **(B)**.

We conducted a non-interventional study at Bordeaux University Hospital between May and July 2018 on a first cohort to test the feasibility of prospectively calculating the IPR. Ninety-one consecutive kidney transplant recipients coming for an outpatient visit between 8 and 16 months post-transplantation were included (Figure 1A). During this inclusion visit, the following patient information was collected: treatments doses, prescription refills and hospitalization stays since transplantation for calculating the IPR, calcineurin inhibitor trough levels, missed outpatient visits, and patient-reported drug side effects. Patients were also asked if they had forgotten to take their medication at least once since transplantation. No pill count was carried out.

#### Second Retrospective Cohort

We tested the association of IPR with the occurrence of clinically apparent histological ABMR (hABMR) in a second retrospective cohort of patients. Between January and December 2020, 451 kidney transplant biopsies were performed at our institution. We excluded 229 biopsies performed during the first 12 months post-transplantation, because the calculation of the IPR required a 6 or 12 months period. Among the 222 remaining for-cause biopsies, we identified 27 patients with a diagnosis of clinical hABMR and 108 with interstitial fibrosis and tubular atrophy (IFTA) without additional specific lesion. The IPR was not available for one ABMR patient because this patient used many community pharmacies and we were not able to recover prescription refills from all of them. The IPR was then compared between the clinical hABMR group (*n* = 26) and an IFTA control group of 26 patients that were matched 1:1 for age and year of transplantation (Figure 1B). In this cohort we compared the IPR-based non-adherence diagnosis with our standard method based on clinical suspicion.

The study was approved by the local Ethics Committee and conducted in accordance with the Declaration of Helsinki. Our clinical database had a French CNIL final agreement, decision 2009-413, n° 1357154, 2 July 2009.

### Measurement of the Immunosuppressant Possession Ratio

**FIGURE 2:**
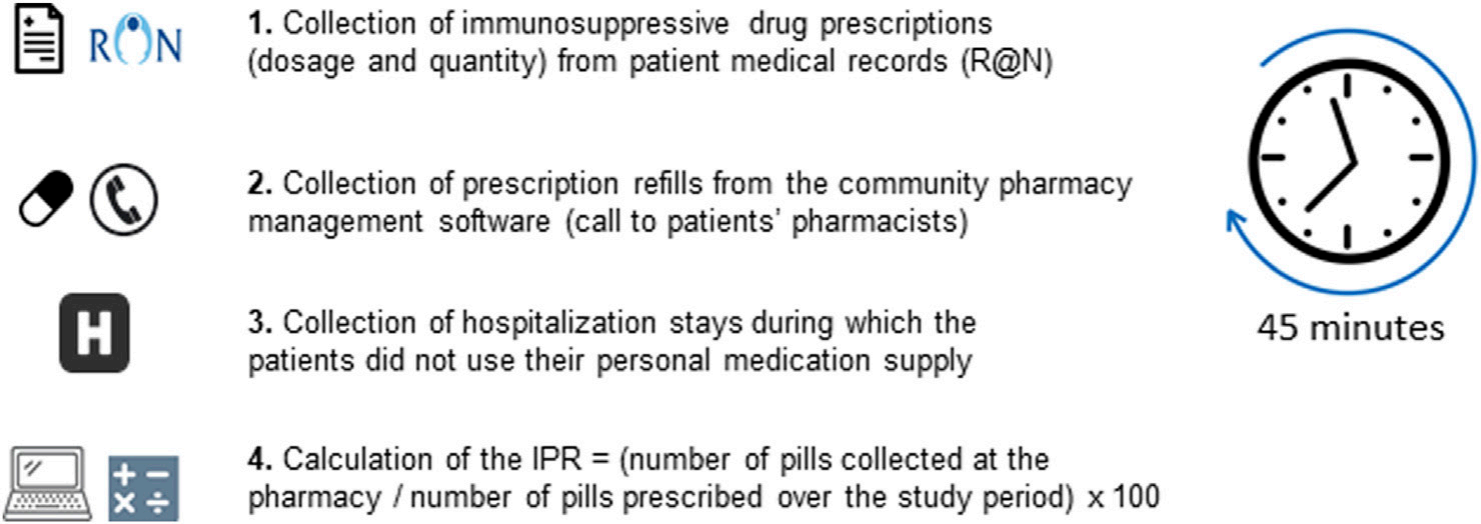
Measurement of the immunosuppressant possession ratio (IPR).

IPR since transplantation was calculated by two transplant clinical pharmacists, as follows: data related to immunosuppressant prescriptions such as dosage and quantity were collected from our patient medical records (R@N); data related to prescription refills and patient dispensing were provided by the patients’ pharmacists using their community pharmacy management software. IPR was then calculated according to the following formula: IPR = (number of pills collected at the pharmacy/number of pills prescribed over the study period) × 100. Importantly, IPR was calculated taking into account hospitalization stays, during which the patients did not use their personal medication supply (Figure 2).

The most frequently used drug for calculating IPR was mycophenolate, in 71 patients (78.0%) in Cohort 1 and 34 patients (65.4%) in Cohort 2, because its dose did not change frequently. If the mycophenolate was discontinued, steroids, everolimus, azathioprine and tacrolimus were used in 17 patients (18.7%), 1 (1.1%), 1 (1.1%) and 1 patient (1.1%), in Cohort 1, respectively, and steroids, azathioprine, tacrolimus and cyclosporine were used in 14 patients (26.9%), 1 (1.9%), 2 (3.8%) and 1 patient (1.9%) in Cohort 2, respectively.

### Measurement of Drug Exposure: Calcineurin Inhibitor Trough Levels

Intra-patient variabilities of tacrolimus and cyclosporine were calculated using the coefficient of variation (CV_TAC_; CV_CsA_). The CV was calculated using the following formula: (standard deviation/mean trough levels of tacrolimus or cyclosporine) × 100. The mean and standard deviation were calculated using all available plasma concentrations. Patients with a CV_TAC_ > 30% were considered to have experienced varying levels of exposure to tacrolimus and were described as being at higher risk of *de novo* DSA and graft loss [30]. In cohort 2, we also measured the last tacrolimus and cyclosporine trough level because values <5 ng/mL, are known to be associated with higher *de novo* DSA incidence [26].

### Clinical Suspicion of Non-Adherence

Clinical suspicion of non-adherence was documented by clinic staff and registered in the patients’ medical records.

### Definition of Histological ABMR

The 222 for-cause biopsies performed were reviewed according to the Banff 2019 classification [16].

We used the term “histological ABMR” (hABMR) proposed by Senev et al. [37] for cases that met the first two Banff 2019 criteria for histology of ABMR. Criterion 1 included one or more of the following lesions: glomerulitis (g), peritubular capillaritis (ptc), arteritis, or thrombotic microangiopathy. Criterion 2 included a microvascular inflammation score (g+ptc) ≥ 2 and/or linear C4d staining on peritubular capillaries [16]. This definition of histological ABMR was made regardless of the third criterion (serological evidence of DSA). Cases with histological ABMR could then be anti-HLA DSA positive or without detectable anti-HLA DSA.

### Identification of Anti-HLA Donor-Specific Antibodies

Sera were tested at the time of each biopsy with single-antigen flow beads assays (SAFB) (One Lambda, Inc., Canoga Park, CA) in accordance with the manufacturer’s recommendations for routine assay use, with ethylenediaminetetraacetic acid in order to avoid the complement interference phenomenon [38–40]. The SAFB were acquired on a Luminex 100^®^ analyzer (Luminex, Austin, TX). Mean fluorescence intensity (MFI) values were normalized using the baseline formula (Fusion^®^ software, One Lambda, Inc.). The positivity threshold was set at MFI ≥ 500.

### Statistical Analysis

The groups were compared using Fisher’s exact test or McNemar’s test for the qualitative variables and Student’s t-test and the Mann–Whitney test for the quantitative variables. The relationship between different computations of the IPR were assessed with Spearman’s correlation (rho). Patient characteristics and pharmacokinetics data were expressed as medians with the interquartile range (IQR). A *p-*value ≤ 0.05 was considered to represent statistical significance. Factors associated with hABMR in cohort 2 were identified using logistic regression. Risk factors with a *p-*value lower than 0.2 in the univariable analysis were included in two multivariable models that were simplified by iterative backward elimination, only keeping the covariables with a *p*-value below or equal to 0.05. A ROC curve analysis was performed to identify an optimal threshold of IPR to predict hABMR. Finally, we used the net reclassification improvement (NRI) to compare the clinical utility of the 6 months IPR < 90% with the clinical suspicion of non-adherence, for the hABMR prediction [41]. The GraphPad Prism v8^®^ software was used for statistical analyses.

## Results

### Prospective Calculation of the Immunosuppressant Possession Ratio

The baseline characteristics of the 91 patients of the first cohort at inclusion are presented in Table 1. All patients received tacrolimus, and 84 (92.3%) were treated with an extended-release formulation (Table 1). Tacrolimus was given in association with mycophenolate in 71 patients (78.0%), everolimus in 12 patients (13.2%) and azathioprine in 2 patients (2.2%). Steroids were given to 62 patients (68.1%).

**TABLE 1:** Patients’ characteristics in prospective cohort 1, according to the immunosuppressant possession ratio.

	All (*n* = 91)	IPR < 90% (*n* = 9)	*p* (vs. IPR = 100%)	IPR = 90–94% (*n* = 6)	*p* (vs. IPR = 100%)	IPR = 95–99% (*n* = 25)	*p* (vs. IPR = 100%)	IPR = 100% (*n* = 51)
Baseline characteristics								
Age (years, IQR)	57 (47–65)	54 (35–71.5)	0.91	60.5 (52–70.3)	0.19	60 (49–64.5)	0.14	55 (43–65)
Female (%)	29 (31.9%)	3 (33.3%)	>0.99	1 (16.7%)	0.65	8 (32.0%)	>0.99	17 (33.3%)
Time since transplantation (months, IQR)	12.7 (10.2–15.6)	13.2 (11.4–15.5)	0.68	17.1 (13.4–17.9)	**0.01**	12.1 (10.1–15.5)	0.82	12.7 (9.6–15.5)
≥2 transplantations (%)	13 (14.3%)	2 (22.2%)	0.66	1 (16.7%)	>0.99	1 (4.0%)	0.15	9 (17.3%)
Hemodialysis (%)	63 (69.2%)	7 (77.8%)	0.71	5 (83.3%)	0.65	17 (68.0%)	>0.99	34 (66.7%)
Peritoneal dialysis (%)	13 (14.3%)	1 (11.1%)	>0.99	1 (16.7%)	>0.99	2 (8.0%)	0.32	9 (17.6%)
Post-transplant educational program (%)	37 (40.7%)	2 (22.2%)	0.29	3 (50.0%)	>0.99	10 (40.0%)	>0.99	22 (43.1%)
Treatment								
Number of medications a day (IQR)	10 (8–13)	9 (8–11)	0.48	11.5 (8–17)	0.50	10 (7–11)	0.42	10 (8–14)
Pillbox use (%)	57 (62.0%)	8 (88.9%)	0.14	3 (50.0%)	>0.99	17 (68.0%)	0.46	29 (56.9%)
Tacrolimus ER (%)	84 (92.3%)	6 (66.7%)	0.06	6 (100%)	>0.99	25 (100%)	0.16	47 (92.2%)
Corticosteroids (%)	62 (68.1%)	7 (77.8%)	>0.99	5 (83.3%)	0.32	14 (56.0%)	0.30	36 (70.6%)
Mycophenolate (%)	71 (78.0%)	5 (55.6%)	0.23	5 (83.3%)	>0.99	22 (88.0%)	0.36	39 (76.4%)
Everolimus (%)	12 (13.2%)	0	0.33	0	0.58	3 (12.0%)	0.74	9 (17.6%)
Azathioprine (%)	2 (2.2%)	0	>0.99	1 (16.7%)	0.20	0	>0.99	1 (2.0%)
Side effects (%)	19 (20.9%)	4 (44.4%)	0.21	1 (16.7%)	>0.99	3 (12.0%)	0.36	11 (22.0%)
Immunosuppressant possession ratio (IPR, median, IQR)	100% (98–100)	85% (76–89)		93% (92–94)		98% (97–99)		100% (100–100)
Tacrolimus exposure								
Tacrolimus trough levels coefficient of variation (CV_TAC,_ median, IQR)	26.2 (20.8–30.7)	32.0 (24.7–36.6)	0.06	27.3 (23.2–28.7)	0.98	22.0 (19.0–28.4)	**0.05**	26.3 (21.5–30.7)
Number of patients with a CV_TAC_ > 30% (%)	26 (28.6%)	6 (66.7%)	**0.05**	1 (16.7%)	0.67	4 (16%)	0.27	15 (29.4%)
Number of patients who claimed having forgotten to take their medicine since transplantation at least once (%)	14 (15.4%)	4 (44.4%)	**0.05**	0	>0.99	3 (13.6%)	>0.99	7 (15.9%)
At least one missed outpatient visit since transplantation (%)	25 (27.5%)	6 (66.7%)	**0.02**	2 (33.3%)	0.65	4 (16%)	0.40	13 (25.5%)

ER, extended-release; IQR, interquartile range; IPR, immunosuppressant possession ratio; SR, standard-release. Quantitative variables are reported as: median (IQR).

Results in bold are the number of patients, column labels and significant p-values (<0,05).

At inclusion, we were able to calculate the IPR since transplantation in all these patients, and the mean time needed to calculate was approximatively 45 min per patient (Figure 2). IPR ranged from 49% to 100% with a median (IQR) of 100% (97–100).

### Immunosuppressant Possession Ratio Since Transplantation Is Associated With Other Markers of Non-Adherence

In the first cohort, patients were divided into three groups according to their IPR (<90%, 90%–94%, 95%–99%) and compared to the patients with an IPR = 100%, in order to determine an optimal non-adherence threshold. Nine patients had an IPR<90% (9.9%) 6 patients an IPR of 90%–94% (6.6%), 25 patients an IPR of 95%–99% (27.5%), and 51 patients an IPR = 100% (56.0%) (Table 1).

Patients with an IPR < 90% had more frequently a CV_TAC_ > 30% (66.7% vs*.* 29.4%, *p* = 0.05), and claimed to have forgotten to take their medication more frequently (44.4% vs*.* 15.9% *p* = 0.05) than patients with an IPR = 100%. Patients with IPRs<90% were also more likely to miss at least one outpatient visit (66.7% vs*.* 25.5%, *p* = 0.02) than patients with IPRs = 100% (Table 1). Patients with an IPR of 95%–99% had a lower CV_TAC_ (22.0% vs 26.3%, *p* = 0.05) than patients with IPRs = 100% (Table 1).

In summary, patients with an IPRs < 90% exhibited more frequently other markers of non-adherence.

### Calculation of the Immunosuppressant Possession Ratio Over a Standardized Period

**FIGURE 3:**
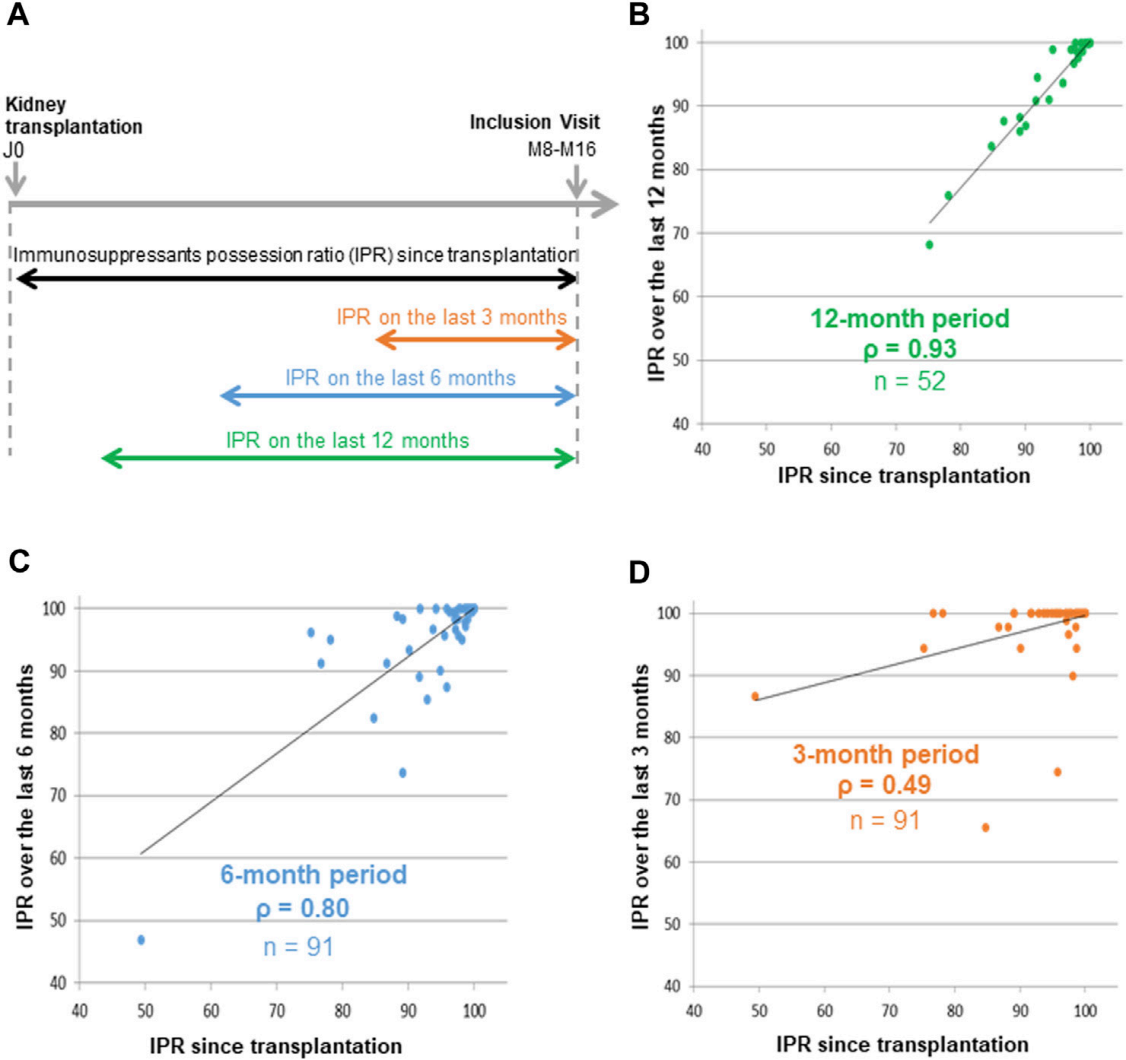
Correlations between immunosuppressant possession ratios since transplantation and three standardized periods The immunosuppressants possession ratio (IPR) was calculated since transplantation for all 91 patients, over the previous 12 months in 52 patients, over the previous 6 months in 91 patients, and over the previous 3 months in 91 patients **(A)**. Spearman correlations were performed between the IPRs calculated since transplantation and the IPRs calculated over the previous 12 months **(B)**, 6 months **(C)** and 3 months **(D)**.

We then tried to determine the optimal duration for calculating the IPR in order to standardize the measurement of this variable. We observed a poor correlation between the IPR calculated over the previous 3 months period and the IPR calculated since transplantation (*ρ* = 0.49). We observed a very good correlation between the IPR calculated over the previous 12 months and the previous 6 months period and the IPR calculated since transplantation (*ρ* = 0.93, and *ρ* = 0.8, respectively) (Figure 3). In summary, the IPR seemed to be calculated reliably over a period of 6 or 12 months. However, the 6 months IPR was used for the rest of the study because it is faster to calculate and more representative of current adherence than the 12 months IPR.

### Six-Month Immunosuppressant Possession Ratio at the Time of Clinical Histological Antibody-Mediated Rejection Diagnosis

We next tested the association of 6 months IPR with the occurrence of clinical hABMR in the retrospective cohort 2. Among 222 for-cause biopsies for the period considered, 26 patients with clinical hABMR were compared to 26 patients with IFTA (Figure 1B). The patients’ characteristics at the time of the for-cause biopsy are depicted in Table 2. No patients had preformed anti-HLA DSA. *De novo* anti-HLA DSA were found in 15/26 patients (57.7%) with clinical hABMR and in 4/26 patients (15.4%) with IFTA (*p* = 0.03).

**TABLE 2:** Patients’ characteristics in retrospective cohort 2.

	All (*n* = 52)	Histological antibody-mediated rejection (*n* = 26)	Interstitial fibrosis and tubular atrophy (*n* = 26)	*p*
Baseline characteristics				
Age (years, median, IQR)	49 (42–62)	50 (41–63)	49 (45–59)	0.89
Female (%)	24 (46.2%)	12 (46.2%)	12 (46.2%)	>0.99
Time since transplantation (months, IQR)	81 (41.3–173.3)	88.5 (41.3–155.3)	72.5 (40.8–192.3)	0.96
Treatment				
Tacrolimus ER (%)	29 (55.8%)	14 (53.9%)	15 (57.7%)	0.78
Tacrolimus SR (%)	8 (15.3%)	5 (19.2%)	3 (11.5%)	0.44
Cyclosporine (%)	13 (25.0%)	6 (23.1%)	7 (26.9%)	0.75
Corticosteroids (%)	35 (67.3%)	19 (73.0%)	16 (61.5%)	0.38
Mycophenolate (%)	34 (65.4%)	17 (65.4%)	17 (65.4%)	>0.99
Everolimus (%)	4 (7.7%)	0	4 (15.4%)	**0.04**
Azathioprin (%)	1 (1.9%)	1 (3.8%)	0	0.31
Sirolimus (%)	1 (1.9%)	1 (3.8%)	0	0.31
Renal injury				
Microvascular inflammation (g + ptc, median, IQR)	1 (0–3)	3 (2–3)	0 (0–0)	**< 0.001**
C4d graft deposition (%)	12 (23.1%)	12 (46.2%)	0	**< 0.001**
Transplant glomerulopathy (cg, median, IQR)	0 (0–2)	1 (0–2)	0 (0–0)	**< 0.001**
Interstitial inflammation and tubulitis (i + t, median, IQR)	0 (0–1)	0 (0–1)	0 (0–1)	0.14
Interstitial fibrosis and tubular atrophy (ct + ci, median, IQR)	4 (2–4)	4 (2–6)	3.5 (2–4)	0.69
Arteriosclerosis (cv, median, IQR)	1 (0–2)	1 (0–2)	2 (1–3)	0.34
*De novo* anti-HLA donor specific antibodies (DSA)				
DSA (%)	19 (38%)	15 (57.7%)	4 (15.4%)	**0.03**
Only class I DSA (%)	1 (1.9%)	1 (3.8%)	0	>0.99
Only class II DSA (%)	10 (19.2%)	7 (26.9%)	3 (11.5%)	0.29
Class I + II DSA (%)	8 (15.4%)	7 (26.9%)	1 (3.8%)	**0.05**
Sum of DSA MFI (arbitrary unit, median, IQR)	0 (0–4,767)	3,542 (0–18,985)	0 (0–0)	**< 0.001**
Treatment exposure				
	***n* = 32** ^a^	***n* = 14**	***n* = 18**	
Last tacrolimus trough level (ng/mL, IQR)	6.3 (5.5–7.9)	6.6 (6.0–8.5)	5.9 (5.1–7.8)	0.29
	***n* = 31** ^b^	***n* = 13**	***n* = 18**	
Tacrolimus trough level coefficient of variation (CV_TAC,_ median, IQR)	24.4 (14.2–34.7)	17.5 (12.8–37.8)	30.0 (16.6–35.2)	0.92
	***n* = 13**	***n* = 6**	***n* = 7**	
Last cyclosporine through level (ng/mL, IQR)	121 (92–152)	103 (65–147)	143 (104–178)	0.23

DSA, donor-specific antibodies; ER, extended-release- IQR, interquartile range-SR, standard-release; MFI, mean fluorescence intensity. Quantitative variables are reported as: median (IQR).

^a^ 15 patients were not treated with tacrolimus and 5 patients had no tacrolimus trough level available on the last year.

^b^ A minimum of three available plasma concentration values was required to calculate the tacrolimus coefficient of variation: incomplete data for one patient.

Results in bold are the number of patients, column labels and significant p-values (<0,05).

**FIGURE 4:**
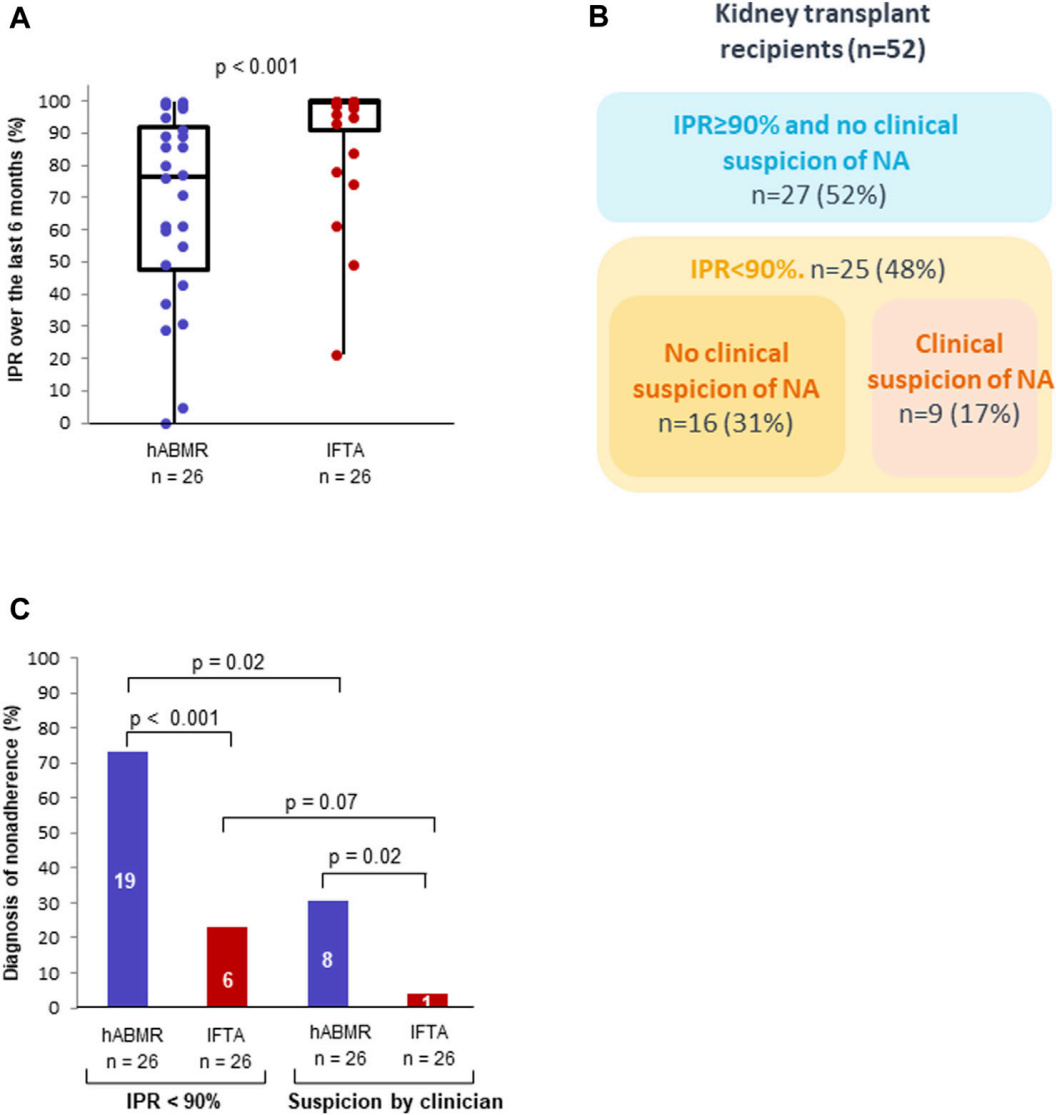
Immunosuppressant possession ratio and clinical suspicion of non-adherence in patients with clinical histological antibody-mediated rejection (hABMR) or interstitial fibrosis with tubular atrophy (IFTA). Description of the immunosuppressant possession ratios calculated over the previous 6 months in patients with a clinical hABMR and IFTA **(A)**. Contingency table of non-adherence identified by immunosuppressant possession ratio <90% over the previous 6 months and clinical suspicion **(B)**. Non-adherence identified by clinical suspicion and immunosuppressant possession ratio <90% over the previous 6 months in patients with a clinical hABMR and IFTA **(C)**.

The 6 months IPR was calculated from the day of the biopsy for the 52 patients. Patients with clinical hABMR had a lower 6 months IPR than patients with IFTA (76% vs*.* 99%, *p* < 0.001) (Figure 4A). Univariable analysis identified only *de novo* DSA and 6 months IPR as risk factors for hABMR. In a first multivariable analysis (model 1), these two variables were independently associated with hABMR (odds ratio, 4.66; 95% CI, 1.19–20.94; *p* = 0.03, and odds ratio, 0.73 per 10% increase; 95% CI, 0.51–0.98; *p* = 0.05, respectively) (Table 3).

**TABLE 3:** Factors associated with histological antibody-mediated rejection.

Variable	Univariable analysis		Multivariable analysis model 1		Multivariable analysis model 2	
	Unadjusted OR (95% CI)	*p*-value	Adjusted OR (95% CI)	*p*-value	Adjusted OR (95% CI)	*p*-value
Age (year)	0.99 (0.96–1.04)	0.84				
Male sex (male *versus* female)	1.00 (0.33–3.00)	>0.99				
Time since transplantation (months)	1.00 (0.99–1.01)	0.96				
Tacrolimus versus cyclosporine	1.23 (0.35–5.51)	0.75				
Mycophenolate versus other	1.00 (0.29–3.46)	>0.99				
Corticosteroids	1.00 (0.58–1.73)	>0.99				
Tacrolimus trough level coefficient of variation >30%	0.58 (0.17–1.93)	0.38				
*De novo* DSA^1, 2^	7.50 (2.15–31.53)	0.003	4.66 (1.19–20.94)	0.03	4.73 (1.17–21.88)	0.03
6-month IPR (10% increase)^1^	0.67 (0.47–0.87)	0.008	0.73 (0.51–0.98)	0.05		
Non-adherence based on 6-month IPR <90%^2^	9.05 (2.72–34.46)	0.0006			6.34 (1.73–25.59)	0.007
Non-adherence based on clinician suspicion^2^	11.11 (1.81–215.6)	0.03				

IPR, immunosuppressant Possession ratio. Covariates with *p*-values < 0.2 on univariable analyses were included into a multiple logistic regression then iteratively removed retaining only those with a *p*-value ≤ 0.05. Variables with the index ^(1)^ were used in the model 1. Variables with the index ^(2)^ were used in the model 2.

### Diagnosis of Non-Adherence Based on 6 Month Immunosuppressant Possession Ratio Below 90%

ROC curve analysis showed that the IPR was a good predictor of hABMR (AUC = 0.79). The optimal predictive threshold of IPR for clinical hABMR occurrence was 92% with a 77% sensitivity and 77% specificity (Supplementary Figure S1). This threshold value was very close to 90% found in cohort 1. Therefore, we chose to compare the diagnosis of non-adherence based on the 6 months IPR < 90% with our standard method based on clinical suspicion. Across the whole cohort, non-adherence was more often diagnosed with the definition based on a 6 months IPR < 90% (25/52 patients) than with the clinical suspicion (9/52 patients) (48.1% vs*.* 17.3%, *p* < 0.001). The diagnosis of non-adherence was achieved by the two methods in 9 KTR (17.3%) and by the IPR < 90% alone in 16 KTR (30.8%). All the patients with a clinical suspicion of non-adherence had an IPR < 90% (Figure 4B).

The proportion of non-adherent KTRs, based on a 6 months IPR < 90% was much higher in the clinical hABMR group (19/26 patients) than in the IFTA group (6/26 patients) (73.1% vs*.* 23.1%, *p* < 0.001) (Figure 4C). In KTRs with clinical hABMR, the percentage of non-adherent KTRs was higher with the definition based on an IPR < 90% (19/26 patients) than with the clinical suspicion (8/26 patients) (73.1% vs*.* 30.8%, *p* = 0.02) (Figure 4C). In KTRs with IFTA, the percentage of non-adherence was also higher with the definition based on an IPR < 90%, but the difference was not significant (23.1% vs*.* 3.8%, *p* = 0.07). Similar results were observed with the 12 months IPR (Supplementary Figure S2).

Univariable analysis also identified 6 months IPR < 90% and non-adherence based on clinical suspicion as risk factors for hABMR (Table 3). In a second multivariable analysis including these two variables and *de novo* DSA (model 2), only *de novo* DSA and 6 months IPR < 90% were independently associated with hABMR (odds ratio, 4.73; 95% CI, 1.17–21.88; *p* = 0.03; and odds ratio, 6.34; 95% CI, 1.73–25.59; *p* = 0.007, respectively).

We finally used the NRI to compare the clinical utility of the 6 months IPR<90% with the clinical suspicion, for the prediction of hABMR. Compared with clinical suspicion, a 6 months IPR < 90% adequately reclassified 42% of patients within the hABMR group, but misclassified 19% of patients of the IFTA group, resulting in a non-significant overall NRI of 0.23 (95% CI −0.07–0.53; *p* = 0.13).

### Immunosuppressant Possession Ratio in Clinical Histological Antibody-Mediated Rejections Related to Anti-HLA DSA

**FIGURE 5:**
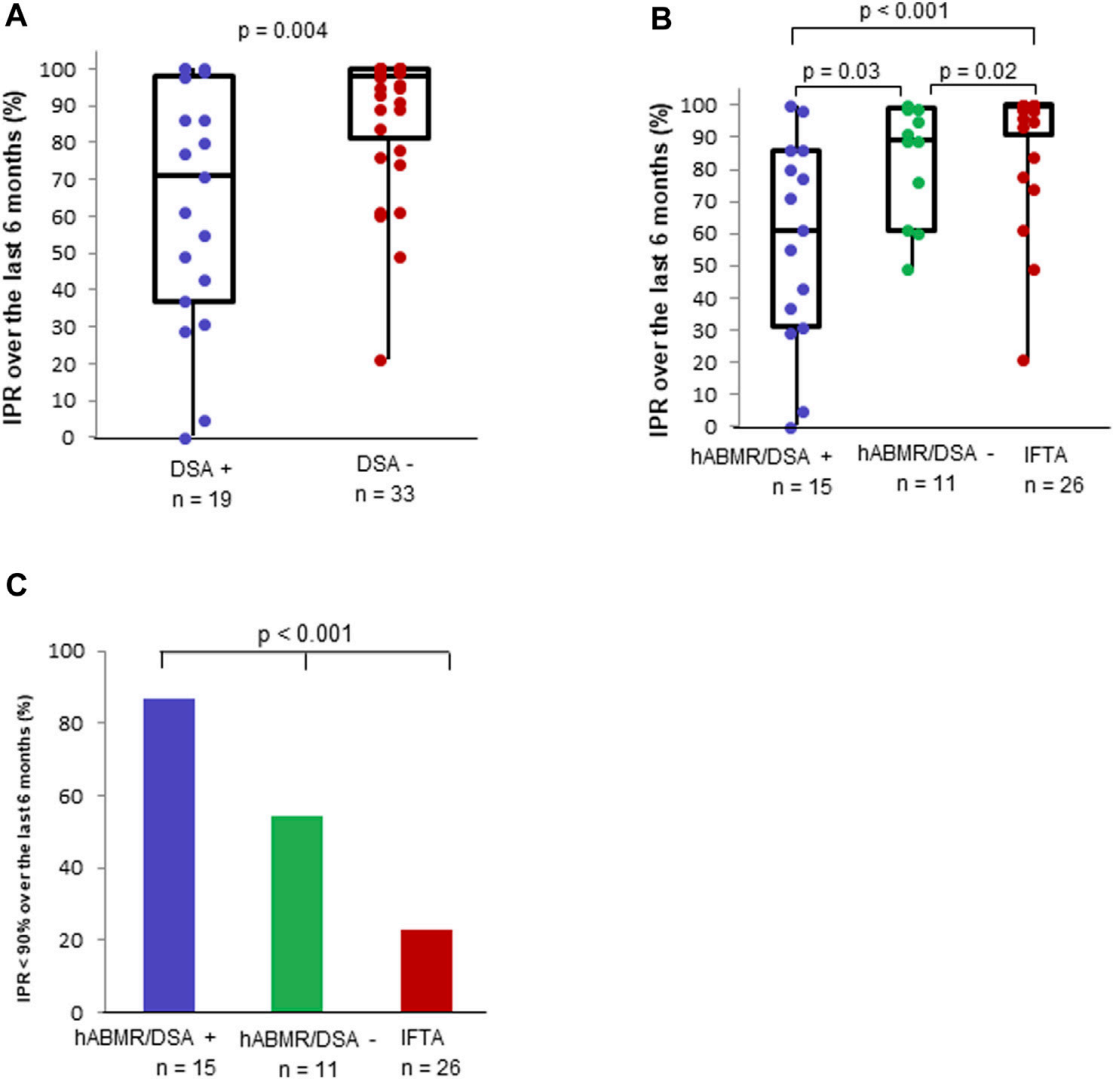
Immunosuppressant possession ratio in patients with or without *de novo* anti-HLA donor-specific antibody (DSA). Description of the immunosuppressant possession ratios calculated over the previous 6 months in patients with or without anti-HLA DSA **(A)**. Description of the immunosuppressant possession ratios calculated over the previous 6 months **(B)** in anti-HLA DSA-positive hABMR, anti-HLA DSA-negative hABMR and IFTA groups. Immunosuppressant possession ratio <90% over the previous 6 months **(C)** in anti-HLA DSA-positive hABMR, anti-HLA DSA-negative hABMR and IFTA groups.

Regardless of histological lesions, *de novo* anti-HLA DSA-positive patients, had a lower 6 months IPR than anti-HLA DSA-negative patients (71% vs*.* 98%, *p* = 0.004) (Figure 5A). Patients with a *de novo* anti-HLA DSA-positive clinical hABMR had a lower 6 months IPR than patients with anti-HLA DSA-negative clinical hABMR (61% vs*.* 89%, *p* = 0.03). Patients with anti-HLA DSA-negative clinical hABMR also had a lower 6 months IPR than patients with IFTA (89% vs*.* 99%, p 0.02) (Figure 5B). Moreover, the proportion of KTRs with a 6 months IPR < 90% was much higher in the anti-HLA DSA-positive clinical hABMR group than in the anti-HLA DSA-negative clinical hABMR and IFTA groups (86.7%, 54.5% and 23.1%, respectively, *p* < 0.001) (Figure 5C). Similar results were observed with the 12 months IPR (Supplementary Figure S3).

## Discussion

This study showed that the prospective calculation of a real-time IPR after transplantation is feasible thanks to a close collaboration between transplant clinical pharmacists and community pharmacists. This IPR was correlated with a high intra-patient variability of tacrolimus trough level and outpatient visit non-adherence. Low IPRs were found at the time of clinical hABMR, especially in patients with *de novo* DSA. This tool was better associated with hABMR than clinical suspicion.

Nowadays, pharmacy management software packages are exhaustive and contain all prescription refill data. These data can be obtained very easily thanks to a close collaboration between the transplant clinical pharmacist and the patient’s pharmacist. By combining them with a reliable collection of each dose change and hospitalization days in the patient’s medical record, it is simple to calculate a very precise IPR for each patient.

We chose to use mycophenolate as a priority for IPR calculation because variations in dosage are infrequent and there are only two commercially available dosages. This method works for brand-name and generic drugs, regardless of the formulation. For those who did not receive mycophenolate, we chose another immunosuppressant with which the calculation of the IPR was simple. We avoided using calcineurin inhibitors because patients must regularly use several different pills of tacrolimus and the dose can vary very frequently. These variations make IPR analysis for calcineurin inhibitors more difficult.

The IPR calculated over a 3 months period was poorly correlated with the IPR calculated for a period of between 8 and 16 months. Some drug packages allow the patient to collect their treatment for 2 or 3 months in a single dispensing. Some patients therefore could have a high IPR over a 3 months period, based on a single dispensing of medication. Choosing to calculate the IPR over a 6 months period seems to be a good compromise because it allows to obtain a reliable, fast and representative calculation of the current adherence.

Patients with IPRs < 90% had higher tacrolimus trough level coefficients of variation compared to patients with IPRs = 100%. This could be explained by a correlation between the mycophenolate possession ratio and the tacrolimus possession ratio. Nevertheless, the latter was not calculated due to its complexity. Patients with an IPR < 90% also claimed to take their medicine less frequently. We also showed that patients with IPRs < 90% were more likely to have had at least one missed visit (66.7% vs. 23.2%, *p* = 0.01). These results are in line with the study of Taber et al. which showed that non-adherence to outpatient visits was strongly correlated with non-adherence to treatment, and both were predictive of adverse clinical consequences [16]. Based on these results, we defined the non-adherent patients as those having a threshold of IPR < 90%. Only 10% of the patients in Cohort 1 had an IPR < 90%. This can be explained by the fact that our patients were adults who had recently been transplanted and because the French health system covers the full cost of immunosuppressants.

It has been reported in previous retrospective studies that non-adherence to immunosuppressants was associated with *de novo* DSA and ABMR [7, 26]. In these studies, non-adherence to immunosuppressants was suspected by transplant physicians. Our study shows that the 6 months IPR < 90% was the only non-adherence measurement tool independently associated with hABMR. It allowed to identify 42% of hABMR patients who had been misclassified by clinical suspicion, confirming the low sensitivity of this latter method [18]. It is also worth noting that measurement of non-adherence with the tacrolimus trough level coefficients of variation was not possible for around half of the patients because they were non-adherent to the recommended biological follow-up. Moreover, the tacrolimus trough level coefficients of variation was not associated with hABMR.

The overall NRI showed only a trend toward a better prediction of hABMR by the IPR < 90% when compared to clinical suspicion. The better identification of hABMR in patients with an IPR < 90% comes at a price of 23.1% of false positive, namely, patients with an IPR < 90% in the IFTA group. A low IPR necessarily implies poor adherence to immunosuppressants, because a patient cannot take treatments he has not collected. Therefore, these 23.1% of false positive patients could be at risk of developing rejection in the future. They may also have acquired operational tolerance, but these two hypotheses deserve to be explored.

The IPR was the lowest in positive anti-HLA DSA-ABMR, but negative anti-HLA DSA-ABMR also had a lower IPR than the control group. Negative anti-HLA DSA-ABMR is an entity caused by non HLA-DSA or missing-self induced microvascular rejection [42, 43]. Our data suggest that non-adherence could also be associated with these recently identified rejections.

One of the limitations of the IPR measurement is that it may be biased if the patient visits different pharmacies without informing medical staff. This phenomenon is rare in France because pharmacies order these expensive treatments only for their usual patients. In addition, patients were asked to report any pharmacy changes. Additionally, patients with IPR = 100% were considered as adherent, but it does not determine whether patients were taking the right dose, even if they had collected their medication from the pharmacy. Another limitation of our study was the small sample size of the two cohorts. However, this did not prevent us from achieving the objectives of the study.

In summary, IPR calculation by transplant clinical pharmacists can be used to diagnose immunosuppressant non-adherence in patients with hABMR. This tool could allow continuous monitoring of adherence and thus take into account the dynamic and individual nature of non-adherence over time. In addition, it could generate a red flag for transplant physicians and pharmacists about patients who are non-adherent to their outpatient visits. Prospective studies are urgently needed to determine its ability to predict all kinds of rejection and graft losses. At the same time, the optimal threshold of IPR associated with the onset of *de novo* DSA and ABMR will have to be determined. An automatic calculation could be envisioned by aggregating the prescription refills which are stored in the national health data system and patients’ medical records in order to save pharmacists time.

## Data Availability

The raw data supporting the conclusion of this article will be made available by the authors, without undue reservation.

## Abbreviations

ABMR, antibody-mediated rejection; CV_TAC_, tacrolimus trough levels coefficient of variation; CV_CsA_, cyclosporine trough levels coefficient of variation; DSA, donor-specific antibodies; g, glomerulitis; hABMR, histological antibody-mediated rejection; IFTA, interstitial fibrosis and tubular atrophy; IPR, immunosuppressant possession ratio; KTR, kidney transplant recipients; ptc, peritubular capillaritis; SAFB, single-antigen flow beads assays.
