# Supplementary figures and images for: Measurement of the Immunosuppressant Possession Ratio by Transplant Clinical Pharmacists Captures a Non-Adherence Associated With Antibody-Mediated Rejection
Source: Transpl Int. 2023 Nov 28;36:11962. doi: 10.3389/ti.2023.11962 (PMC10713790; doi:10.3389/ti.2023.11962)

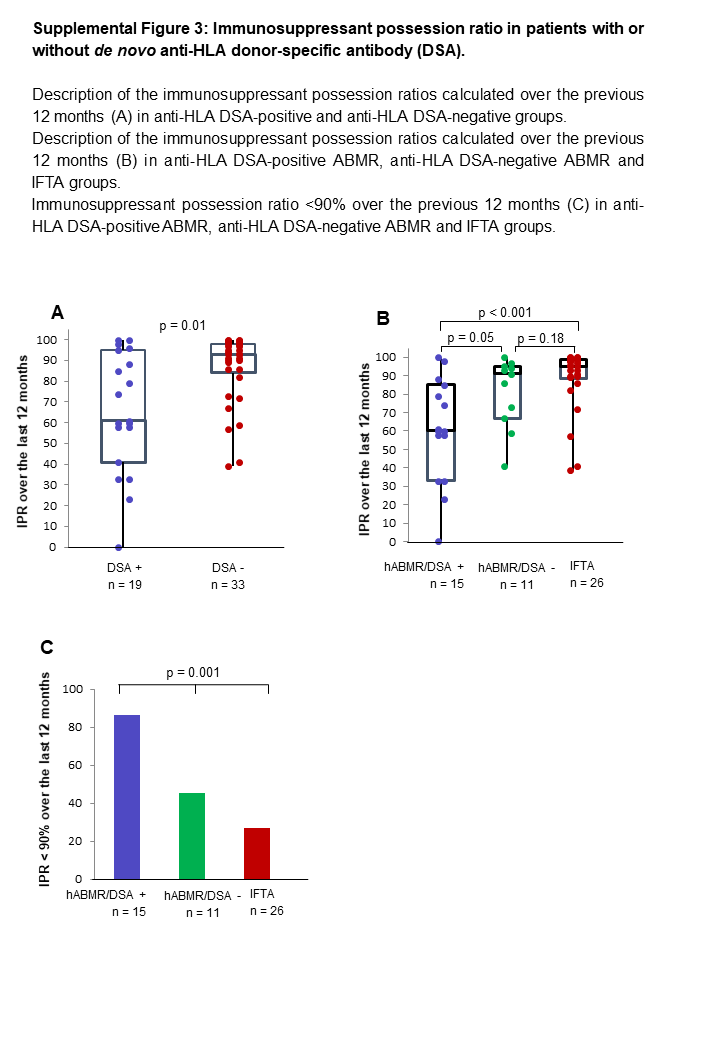

Supplement: Supplementary file 1 [file Image3.TIF]

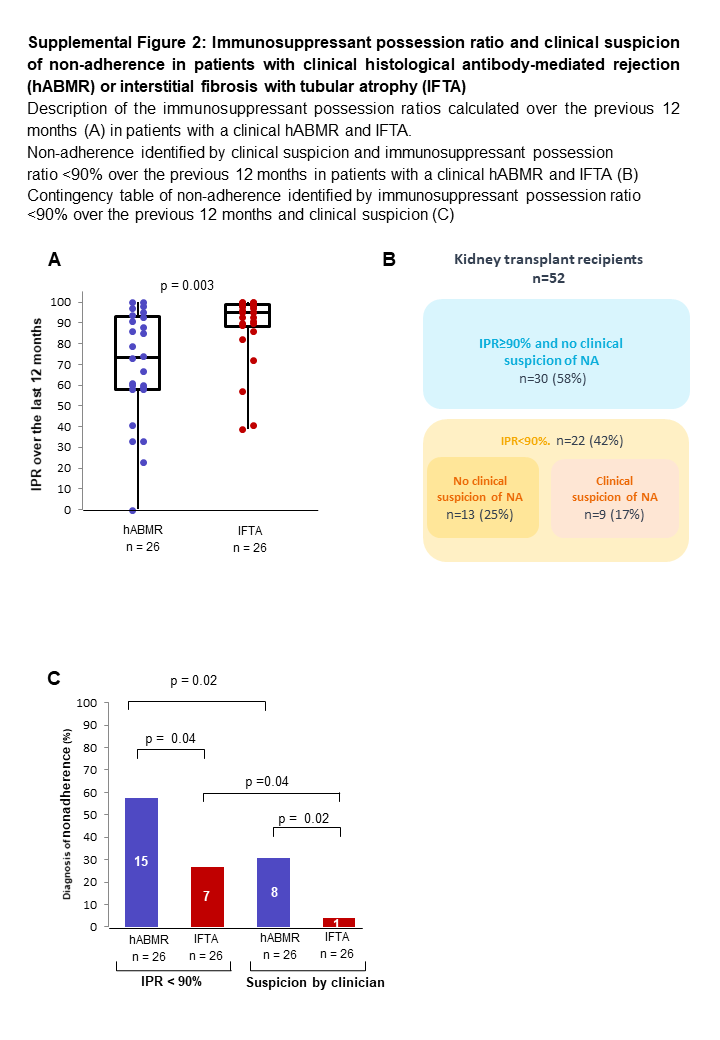

Supplement: Supplementary file 2 [file Image2.TIF]

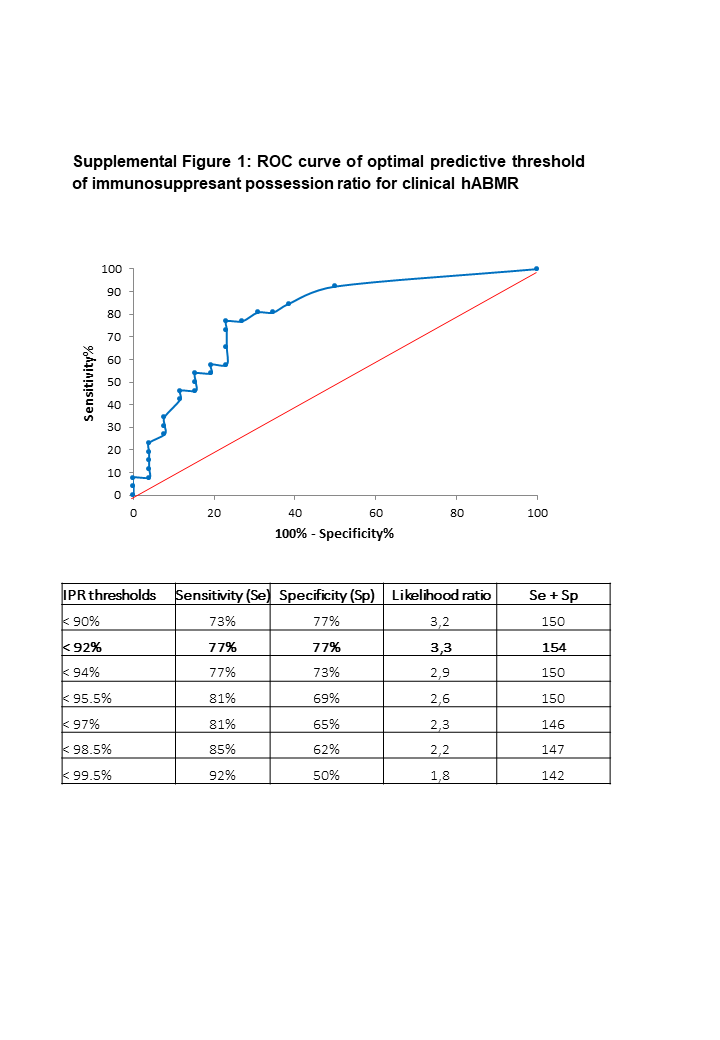

Supplement: Supplementary file 3 [file Image1.TIF]
